# Delayed Surgical Intervention for a Self-Inflicted Left Chest Injury From a Nail Gun: Addressing 28 Nails Six Months Post-incident

**DOI:** 10.7759/cureus.56789

**Published:** 2024-03-23

**Authors:** Omar Eldeib, Ahmed Eldeib, Loren Harris

**Affiliations:** 1 Surgery, State University of New York (SUNY) Downstate Health Sciences University College of Medicine, Brooklyn, USA; 2 Surgery, Richmond University Medical Center, Staten Island, USA

**Keywords:** hemoptysis, robotic lung resection, thoracotomy, trauma, foreign body

## Abstract

In 2021, there were 1.7 million suicide attempts in the US, with firearms being the most common method used, comprising more than 50% of attempts. We present a case report of delayed operative intervention for foreign body removal after a suicide attempt using a nail gun fired into the left hemi thorax. Initial conservative management was complicated by the delayed presentation of hemoptysis requiring surgical intervention. We hypothesize that the delay was secondary to foreign body migration. Initial management was attempted robotically, but dense adhesions were encountered, requiring conversion to an open thoracotomy. Twenty-eight nails were removed. The post-operative course was uncomplicated.

## Introduction

In 2021, there were 1.7 million suicide attempts in the US, with firearms being the most common method used, comprising more than 50% of attempts [1]. Self-inflicted penetrating thoracic injuries are a common cause of death in suicide attempts. Mortality from penetrating thoracic injuries varies in the literature, with certain reports quoting up to 79% [2].

Nail guns are tools that were developed to help drive nails into various materials with ease using several different mechanisms including electric, pneumatic, and combustion-based actuated mechanisms [3]. Steel nails are the most common type of nails used in construction and other applications [4]. Nail guns are not considered “firearms” according to federal law however nail guns fire nails with substantial force, enough to cause serious harm and sometimes even cause death [5].

## Case presentation

A 46-year-old male with a history of post-traumatic stress disorder (PTSD) presented to our level 1 trauma center as a level 1 activation code after sustaining a self-inflicted nail gun injury to his left chest in a suicide attempt. The family found the patient in a pool of blood with a nail gun beside the patient and an empty bottle of Clonazepam. The patient was brought to the hospital within one hour of injury.

**Figure 1:**
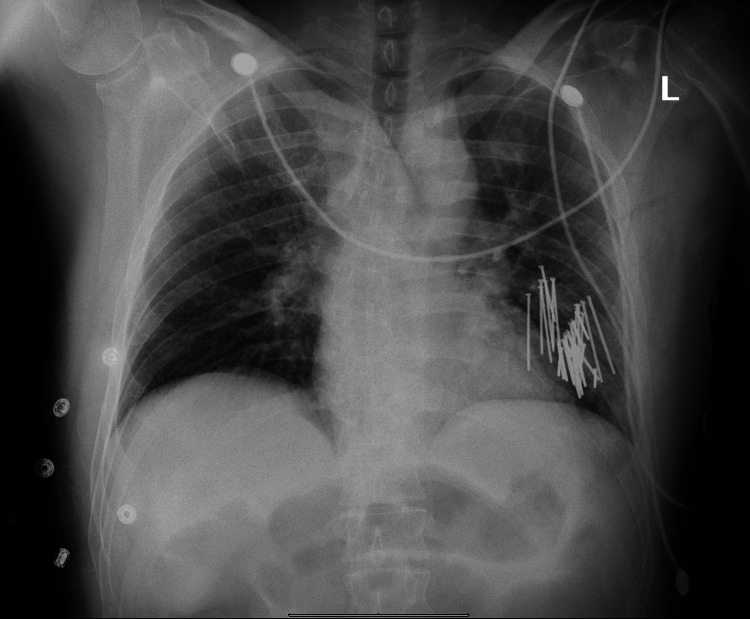
Chest x-ray upon initial presentation

On arrival, the patient was tachycardic to 115 normotensives, however, was obtunded. The patient was intubated for airway protection. On physical exam, one small puncture wound was appreciated and there was a large amount of subcutaneous emphysema of the left anterior chest wall. A chest x-ray (Figure 1) was obtained and showed multiple nails in the left lung and no substantial pneumothorax was appreciated. Based on the mechanism of injury, and to prevent deterioration with positive pressure ventilation in case a small occult pneumothorax was present a 28Fr tube thoracostomy was performed in the trauma bay with 40cc of blood output.

**Figure 2:**
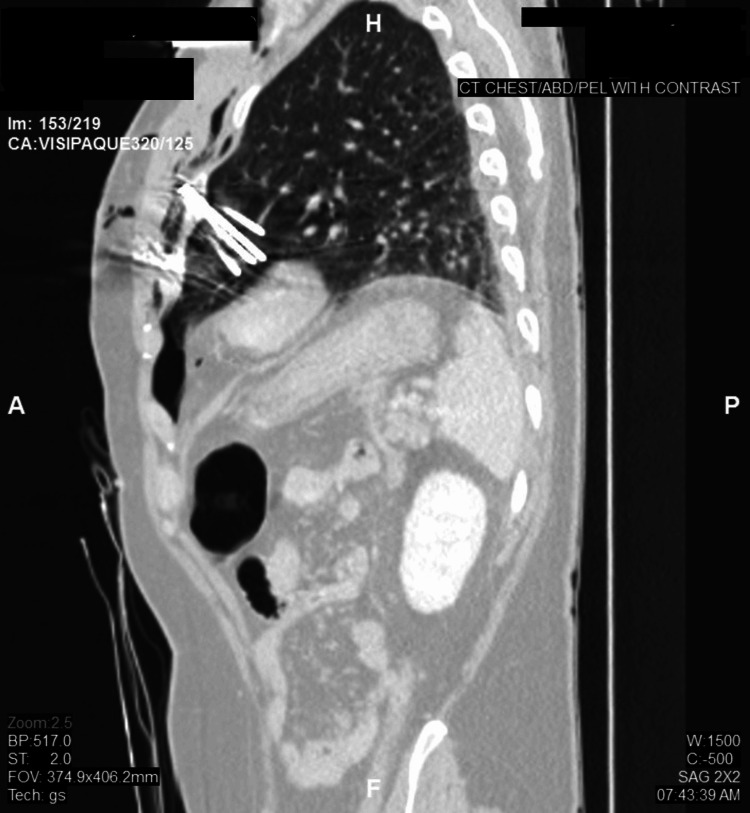
CT angiogram of chest

The rest of the primary and secondary surveys were unremarkable. E-FAST was deferred. The patient then underwent a chest and abdomen CT angiogram (Figure 2) which showed multiple nails imbedded in the left lung, with a nail that was a few millimeters away from the anterior wall of the heart and the pericardium. No pericardial effusion was noted, and no vascular injury was reported. Small apical pneumothorax and pneumomediastinum were appreciated.

The patient remained stable and was transferred to the Surgical Intensive care unit (SICU) for close observation. The decision was made not to remove the nails as the patient was stable and the nails did not damage any vital structures.

The patient was seen by an inpatient psychiatric service and was started on antipsychotics. On hospital day 2, his chest tube was removed, and post-removal chest x-ray did not show the formation of pneumothorax and subsequent daily chest x-rays remained unchanged.

The patient was downgraded to the floor on hospital day 4 and after being cleared by inpatient psychiatry, he was discharged home on hospital day 9 with a close follow-up in the trauma clinic. The patient was seen in the trauma clinic about one month later, and his only complaint was mild intermittent chest discomfort with deep inspiration.

**Figure 3:**
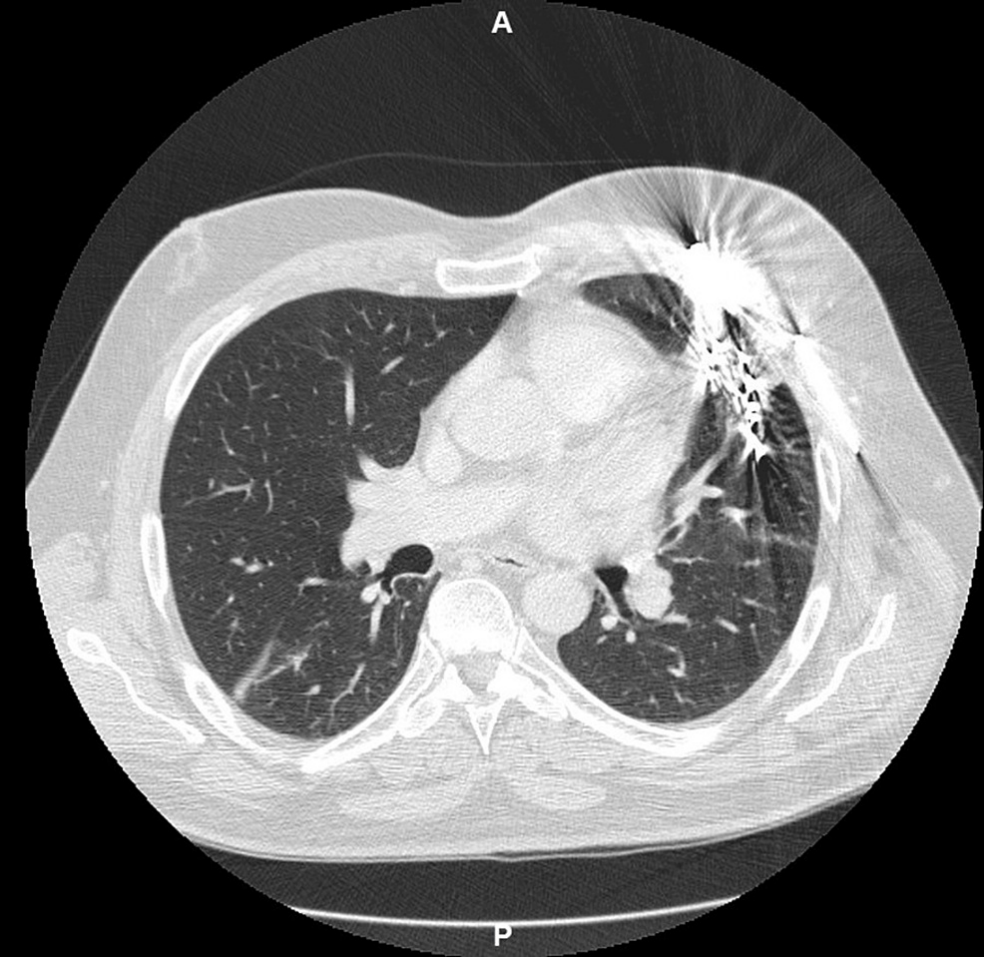
Repeat chest CT scan

The patient returned to the thoracic surgery clinic six months later with complaints of one episode of hemoptysis and chest discomfort. A repeat chest CT scan (Figure 3) showed that the nails had migrated and were in very close proximity to the lingular artery of the left upper lobe. No extravasation was noted on imaging. This was presumed to be the source of the hemoptysis and the patient was scheduled for a robotic resection of the left upper lobe lingular segment and removal of the parenchymal foreign bodies.

**Figure 4:**
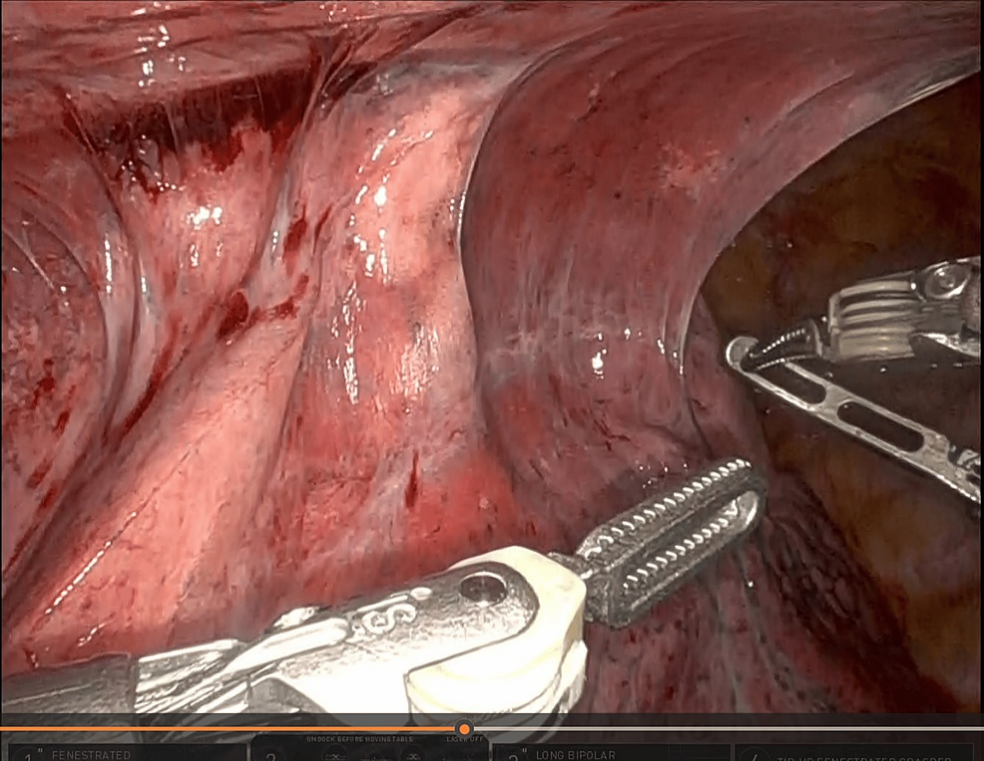
Thoracoscopic view

**Figure 5:**
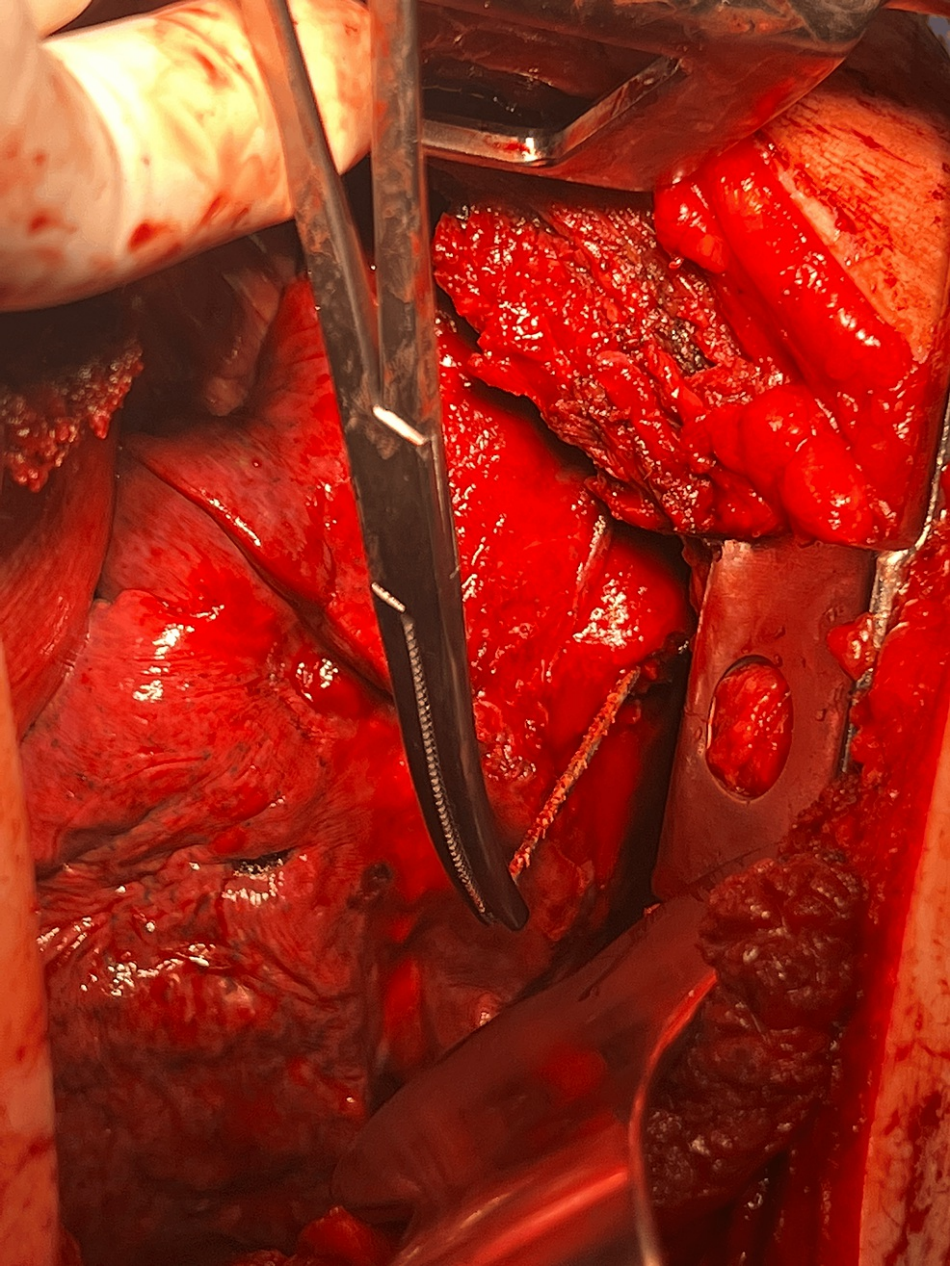
Open view during extraction

Upon entering the chest cavity dense adhesions were appreciated between the lung, the pleura, pericardium, and the anterior chest wall (Figure 4). Meticulous dissection was performed however dissection was very challenging due to the dense fibrous adhesions with only partial mobilization of the left upper lobe. Bleeding from the pericardial fat and surrounding fibrous tissue made further robotic progress unlikely and the decision was made to convert to open. A left posterolateral thoracotomy at the fifth intercostal space was performed. Hemostasis was achieved. Due to the fact that there was no dissection plane between the medial portion of the lingua, the pericardium and the chest wall, a non-anatomic lingular resection was performed. The remaining intra-parenchymal nails could be easily palpated, and each nail was manually removed by using electrocautery to open the lung tissue at the tip of each nail with subsequent extraction using a Kelly Clamp (Figure 5). This allowed for the preservation of the surrounding healthy lung tissue.

**Figure 6:**
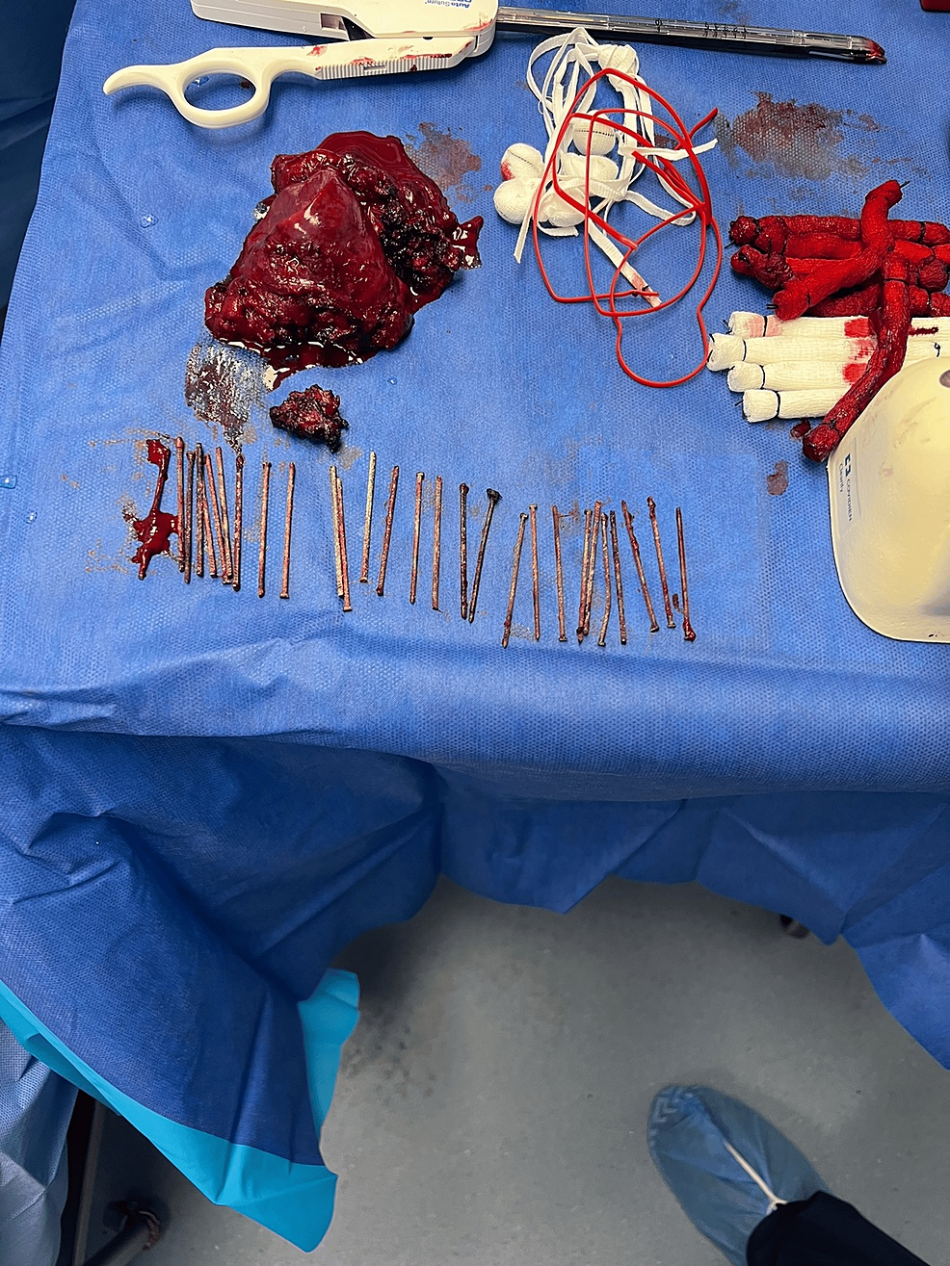
Extracted nails

There were still a few embedded nails in the anterior chest wall which were left in place. A total of 28 nails were removed (Figure 6). A 28 Fr chest tube was placed, and the chest was closed in standard fashion. Estimated blood loss was about 3L and the patient received six units of packed red blood cells and three units of fresh frozen plasma. The bleeding was predominantly lung parenchymal bleeding due to a lack of surgical planes due to extensive fibrosis.

There was minimal post-operative air leak from the chest tube it was removed on post-operative day 3. The patient was eventually discharged on post-operative day 5.

## Discussion

Unintentional injuries using nail guns are not uncommon. However, suicide attempts using these guns are rare with few such cases reported in the literature. A large number of published literatures favors conservative management of retained pulmonary foreign bodies in patients without symptoms [6]. One study showed that only 13% of patients with retained pulmonary parenchymal foreign bodies ultimately required surgical removal [7]. Once symptoms such as infection or bleeding occur, surgical excision or removal should be considered [8]. The occurrence of hemoptysis prompted a repeat chest CT with IV contrast which showed the migration of the intra-parenchymal nails into the lingular segment of the upper lobe with proximity to the lingular branch of the pulmonary artery. The assumption was made that this may have been responsible for the patient’s hemoptysis and thus was an indication for surgical extraction. Foreign body migration is a well-known phenomenon and might be related to gravity, local erosion, coughing, and increased risk of complications [6,9]. The extreme extent of inflammatory reaction caused by these intra-parenchymal nails which led to the need for conversion to thoracotomy was unforeseen and has not been previously reported. Perhaps knowing that would occur could make a case for earlier surgical intervention which would have a higher chance of being completed successfully using minimally invasive techniques.

## Conclusions

While trauma literature may typically advocate for conservative management of pulmonary foreign bodies, the significant ballistic burden reported above underscores the necessity of carefully considering early surgical intervention in specific cases, as delayed removal can result in extensive fibrosis, emphasizing the importance of such considerations in surgical planning.
